# Meiotic Recombination in Arabidopsis Is Catalysed by DMC1, with RAD51 Playing a Supporting Role

**DOI:** 10.1371/journal.pgen.1003787

**Published:** 2013-09-26

**Authors:** Olivier Da Ines, Fabienne Degroote, Chantal Goubely, Simon Amiard, Maria E. Gallego, Charles I. White

**Affiliations:** Génétique, Reproduction et Développement, UMR CNRS 6293 - Clermont Université - INSERM U1103, Aubière, France; University of Birmingham, United Kingdom

## Abstract

Recombination establishes the chiasmata that physically link pairs of homologous chromosomes in meiosis, ensuring their balanced segregation at the first meiotic division and generating genetic variation. The visible manifestation of genetic crossing-overs, chiasmata are the result of an intricate and tightly regulated process involving induction of DNA double-strand breaks and their repair through invasion of a homologous template DNA duplex, catalysed by RAD51 and DMC1 in most eukaryotes. We describe here a RAD51-GFP fusion protein that retains the ability to assemble at DNA breaks but has lost its DNA break repair capacity. This protein fully complements the meiotic chromosomal fragmentation and sterility of Arabidopsis *rad51*, but not *rad51 dmc1* mutants. Even though DMC1 is the only active meiotic strand transfer protein in the absence of RAD51 catalytic activity, no effect on genetic map distance was observed in complemented *rad51* plants. The presence of inactive RAD51 nucleofilaments is thus able to fully support meiotic DSB repair and normal levels of crossing-over by DMC1. Our data demonstrate that RAD51 plays a supporting role for DMC1 in meiotic recombination in the flowering plant, Arabidopsis.

## Author Summary

Recombination ensures coordinated disjunction of pairs of homologous chromosomes and generates genetic exchanges in meiosis and, with some exceptions, involves the co-operation of the RAD51 and DMC1 strand-exchange proteins. We describe here a RAD51-GFP fusion protein that has lost its DNA break repair capacity but retains the ability to assemble at DNA breaks in the plant, Arabidopsis - fully complementing the meiotic chromosomal fragmentation and sterility of *rad51* mutants, and this depends upon DMC1. No effect on genetic map distance was observed in complemented *rad51* plants even though DMC1 is the only active strand transfer protein. The inactive RAD51 nucleofilaments are thus able to fully support meiotic DSB repair and normal levels of crossing-over by DMC1 in Arabidopsis. The RAD51-GFP protein confers a dominant-negative inhibition of RAD51-dependent mitotic recombination, while remaining fully fertile - a novel and valuable tool for research in this domain. These phenotypes are equivalent to those of the recently reported yeast *rad51-II3A* mutant, (Cloud et al. 2012), carrying the implication of their probable generality in other eukaryotes and extending them to a species with a very different relation between numbers of meiotic DNA double-strand breaks and crossing-overs (∼2 DSB/CO in yeast; ∼25–30 DSB/CO in Arabidopsis; ∼15 DSB/CO in mice).

## Introduction

Meiosis is the specialised cell division essential for sexual reproduction that halves the chromosome number in the production of gametes. It is characterised by one round of DNA replication followed by two successive divisions, resulting in the production of 4 haploid nuclei from a single mother cell. In contrast to the mitotic cell divisions of development and growth, meiosis necessitates the recognition and coordinated segregation of pairs of homologous chromosomes, a function ensured by meiotic recombination in the majority of studied eukaryotes (reviews by [1], [2]).

Meiotic recombination is initiated by programmed DNA double strand breaks (DSBs), which are resected to generate 3′ single-stranded DNA overhangs (ssDNAs) that are bound by specialised recombinases. The resulting nucleoprotein filaments catalyse the invasion of a homologous DNA template by the 3′-ended DNA strand(s) to form a joint recombination intermediate, which in turn can be processed to yield crossing-over (CO) or non-crossing-over (NCO) products. In most eukaryotic organisms, the crucial invasion step of meiotic recombination requires the co-operation of the RAD51 and DMC1 recombinases. Biochemical and structural analyses indicate that RAD51 and DMC1 have homologous DNA pairing and strand exchange activities and have similar properties [3]–[7]. However, DMC1 is only required in meiosis while RAD51 is essential for both mitotic and meiotic recombination [8]–[11].

Repair of mitotic DSB is believed to principally involve the invasion of the sister chromatid, while during meiosis both sister and non-sister chromatids serve as templates for repair [12]–[15]. The choice of template for repair of DSBs is a key and specific feature of meiosis and must be tightly regulated to favour interhomologue recombination and crossing-over that ensure coordinated chromosomal disjunction at the first meiotic anaphase [8], [13], [16]. The RAD51 and DMC1 recombinases play key roles in these events and DMC1 is specifically implicated in meiotic interhomologue crossing-over [16].

Budding yeast RAD51 and DMC1 proteins share both overlapping and distinct functions during meiotic recombination [8], [17]–[19]. Absence of RAD51 strongly affects meiotic recombination and results in failure to repair DSBs and cell cycle arrest. Lack of DMC1 leads to a similar phenotype, with *dmc1* mutants producing some viable spores [8], [20] and these defects of *dmc1* mutant cells can be partially complemented by overexpression of RAD51 [21]. DMC1 nucleofilament formation is altered in the *rad51* mutant, but RAD51 localisation appears normal in *dmc1* mutants [17]. Simultaneous mutation of both recombinases results in a more severe phenotype than either of the single mutants [18], [19]. Both RAD51 and DMC1 are indispensable for efficient meiotic recombination in yeast and given similar activities of the two proteins, it has been generally accepted that they play similar roles in catalysing the invasion of the template DNA duplex. This assumption has however been called into question by the recent characterisation of the catalytically inactive yeast *rad51-II3A* mutant, showing that it is the presence of the RAD51 protein and not its strand-exchange activity that is needed in meiosis [22], [23]. In contrast, the equivalent *dmc1-II3A* mutant protein is inactive and has the same meiotic prophase arrest and absence of joint-molecule formation as the *dmc1Δ* mutant.

Given the importance of DMC1 for meiotic crossing-over and the considerable variation in the ratios of numbers of meiotic DSB and CO in different organisms, it is possible that the situation is more complex in vertebrates and higher plants (about 15 or 25 DSB per CO in mouse or Arabidopsis versus 2 in budding yeast (reviewed by [24]). Mouse *dmc1* knockout mutants are completely sterile, with defects in homologous chromosome pairing, synapsis and DSB repair [25], [26] but the lethality of the *rad51* mutants in vertebrates has hampered the study of their meiotic phenotype. Arabidopsis *rad51* and *dmc1* mutants have strikingly different meiotic phenotypes [27]–[32]. The chromosomes of *rad51* mutants fragment in late meiotic prophase I and the plants are completely sterile [28]. In contrast and notwithstanding the absence of chiasmata and bivalents, meiotic chromosomes of *dmc1* mutant plants remain intact and the plants have some fertility (∼1.5%; Couteau et al., 1999). As for yeast, loading of DMC1 is strongly reduced in Arabidopsis *rad51* mutants, however localisation of RAD51 on meiotic chromosomes appears not to depend upon DMC1 [31], [32].

These differing meiotic phenotypes of Arabidopsis *rad51* and *dmc1* mutants have been generally accepted to be a clear illustration of DMC1 driving meiotic DSB repair through non-sister chromatid donors, while RAD51-driven repair uses sister-chromatid donors (discussed by [2]). An interpretation called into question by this work. We present here the analysis of a novel Arabidopsis RAD51 separation-of-function mutant, showing that RAD51 plays an essential role in supporting the activity of DMC1, which alone is sufficient to promote full homologous pairing, crossing-over and DSB repair in Arabidopsis meiosis.

## Results

### Construction of a RAD51-GFP translational fusion and functional verification by complementation analysis

**Figure 1:**
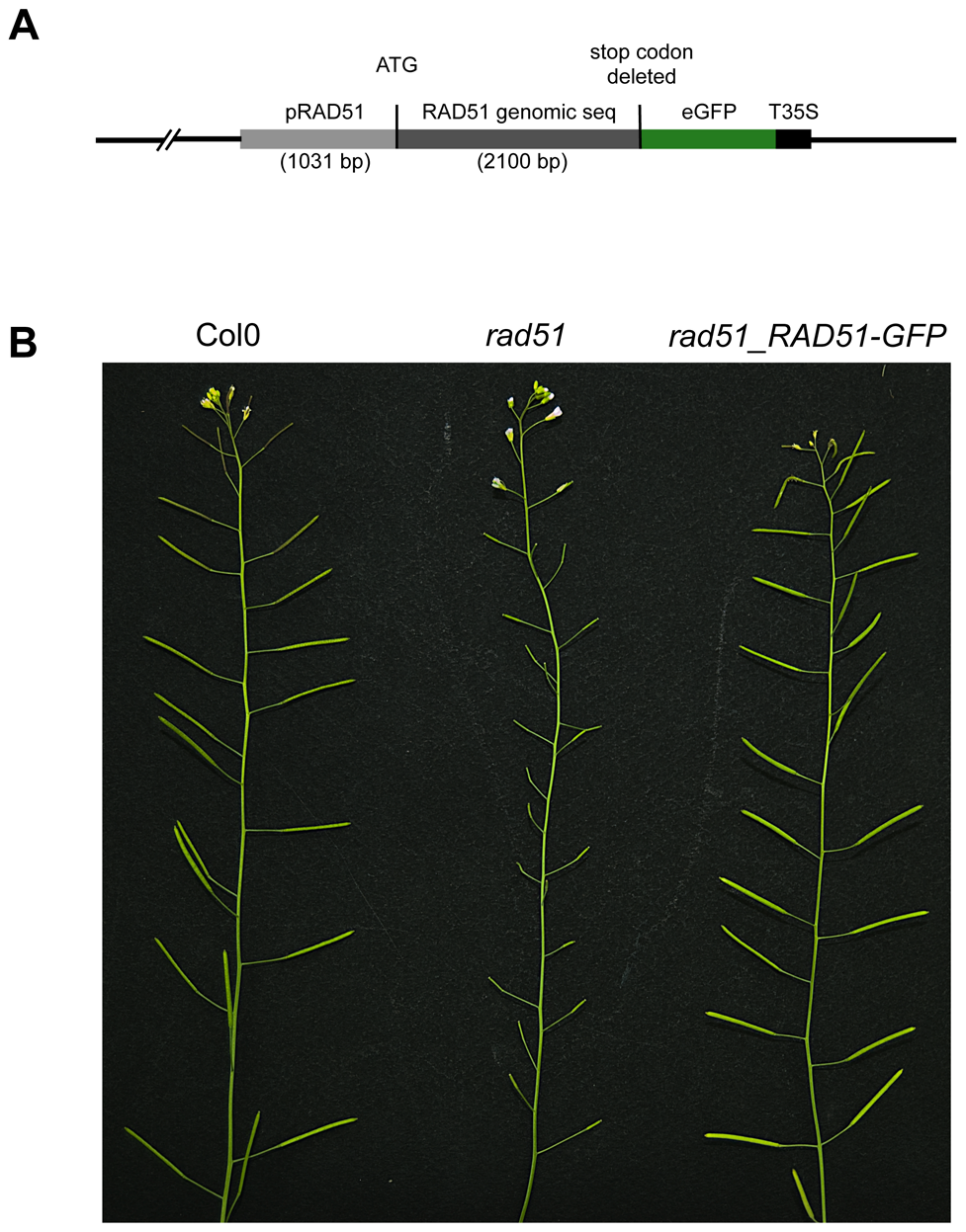
RAD51-GFP restores fertility of the Arabidopsis *rad51* mutant. (**A**) Schematic representation of the RAD51-GFP fusion construct. (**B**) Wild-type plants have long siliques full of seeds, while *rad51* mutants are completely sterile. Expression of RAD51-GFP in *rad51* mutants restores fertility.

RAD51 plays a central role in homologous recombination (HR) in both mitotic and meiotic cells of eukaryotes, including plants. To further investigate the roles of this protein during homologous recombination *in planta*, we constructed a RAD51-GFP translational fusion (Figure 1A). The *RAD51* genomic coding sequence, including introns but without the stop codon, and 1031 bp of upstream sequence was amplified by PCR from DNA of wild-type Arabidopsis (Columbia) and the eGFP coding sequence fused to the 3′ end of the *RAD51* open reading frame (Figure 1A). The fusion construct was introduced into *RAD51/rad51* heterozygote plants and transformants expressing the RAD51-GFP translational fusion protein were selected. PCR genotyping of the *RAD51* locus of the 32 RAD51-GFP transformants showed that 5 were *rad51/rad51*, 19 *RAD51/rad51* and 8 were *RAD51/RAD51*. All five *rad51/rad51* plants expressing the RAD51-GFP translational fusion protein were fully fertile, confirming that the fusion protein is able to complement the sterility phenotype of the Arabidopsis *rad51/rad51* mutant (Figure 1B). This complementation strictly cosegregated with the transgene in the following generation. The RAD51-GFP fusion protein is thus properly expressed and functional during meiosis in these plants.

### Wild-type meiotic progression in RAD51-GFP complemented plants

**Figure 2:**
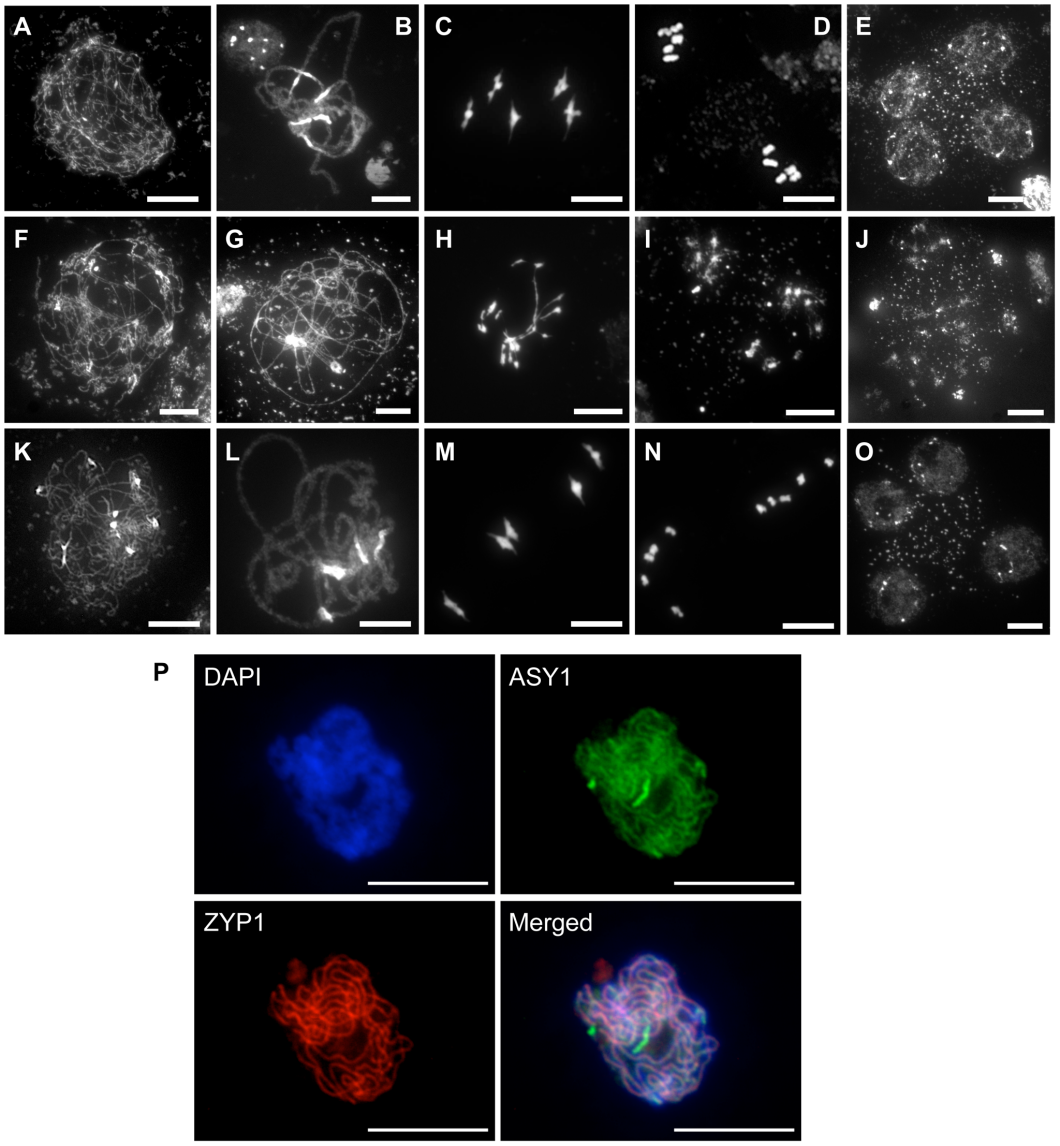
Progression of meiosis in WT (A to E), *rad51/rad51* (F to J) and *rad51/rad51* plants expressing the RAD51-GFP fusion protein (K to O). DAPI staining of pollen mother cells (PMC) during meiosis. (**A, F, K**), Leptotene, (**B, G, L**) Pachytene, (**C, H, M**) Metaphase I, (**D, I, N**) Metaphase II and tetrad showing four meiotic products (**E,O**) and the equivalent stage with heterogeneous DNA masses in the rad51 mutant (**J**). (Scale Bar: 10 µm). (**P**) Immunolocalisation in *rad51/rad51* RAD51-GFP PMC shows that synaptonemal complex (SC) proteins ASY1 (SC axial element) and ZYP1 (SC transverse filament) are correctly loaded along chromosome axes at pachytene indicating normal completion of synapsis. DAPI (blue), ASY1 (green), ZYP1 (red) and merged images are shown. (Scale Bar: 10 µm).

The fertility of the *rad51/rad51* mutant plants complemented by the fusion protein clearly establishes that RAD51-GFP is able to substitute for the RAD51 protein in its essential meiotic role. A detailed cytogenetic analysis of meiotic progression in pollen mother cells (PMC) of plants expressing the RAD51-GFP fusion protein confirmed this, with meiotic stages appearing indistinguishable from wild-type meiosis (Figure 2). In wild-type plants, meiotic chromosomes condense at leptotene (Figure 2A). Pairing and synapsis of homologues is seen as the synaptonemal complex at pachytene (Figure 2B). Chromosomes further condense and the expected five bivalents are observed at metaphase I (Figure 2C). Homologous chromosomes then segregate to opposite poles to give two sets of five chromosomes at metaphase II (Figure 2D). Meiosis II then proceeds and gives rise to 4 haploid nuclei (Figure 2E). In contrast, pairing and synapsis are strongly impaired in *rad51/rad51* mutant (Figure 2G). Defects in DSB repair further lead to strong chromosome fragmentation, fusion and chromosome mis-segration producing unbalanced and fragmented polyads (Figure 2H–J). In *rad51/rad51* RAD51-GFP plants, meiosis appears indistinguishable from wild-type resulting in the expected 4 haploid meiotic products (Figure 2K–O). Normal structure of the synaptonemal complex at pachytene of *rad51/rad51* RAD51-GFP meiosis was confirmed by immunolocalisation of the synaptonemal complex (SC) axial element protein ASY1 [33] and the SC transverse filament protein ZYP1 [34] (Figure 2P).

### RAD51-GFP is inactive in mitotic recombination and confers a dominant-negative phenotype

**Figure 3:**
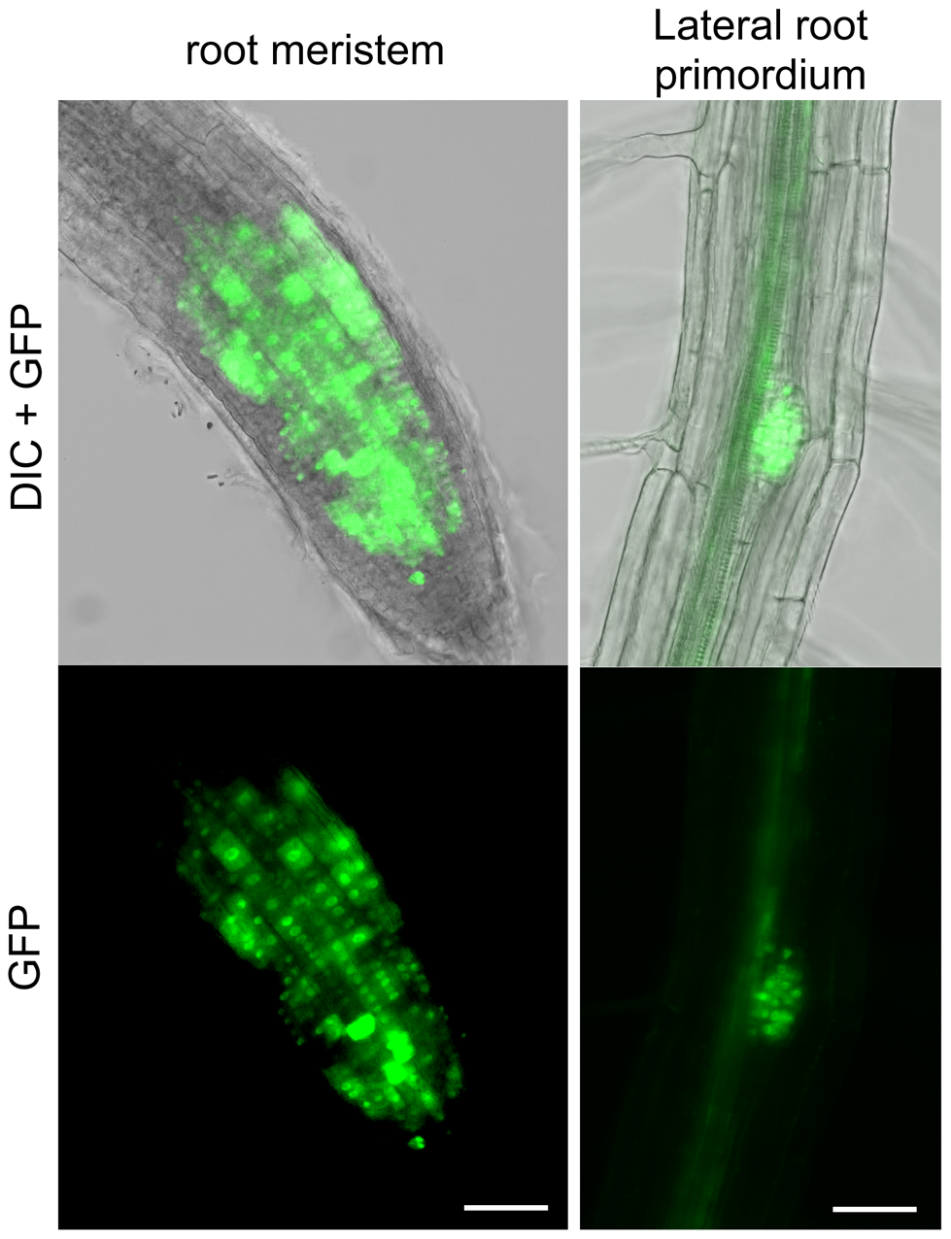
RAD51-GFP is expressed in nuclei of meristematic cells in primary and lateral roots. RAD51-GFP fusion protein is present in the nuclei of primary root meristem (left panels) and lateral root meristem cells (right panels). No or faint signals are observed in transition and elongation zone. Differential interference contrast (DIC)+GFP (upper panels) and GFP fluorescence (lower panels) images are shown. (Scale bar = 50 µm.).

As seen above, the RAD51-GFP fusion protein is properly expressed and functional during meiosis. In somatic tissues, the expected strong GFP expression is visible in nuclei of meristematic cells in primary and lateral roots and none detected in non-dividing root transition and elongation zones (Figure 3).

**Figure 4:**
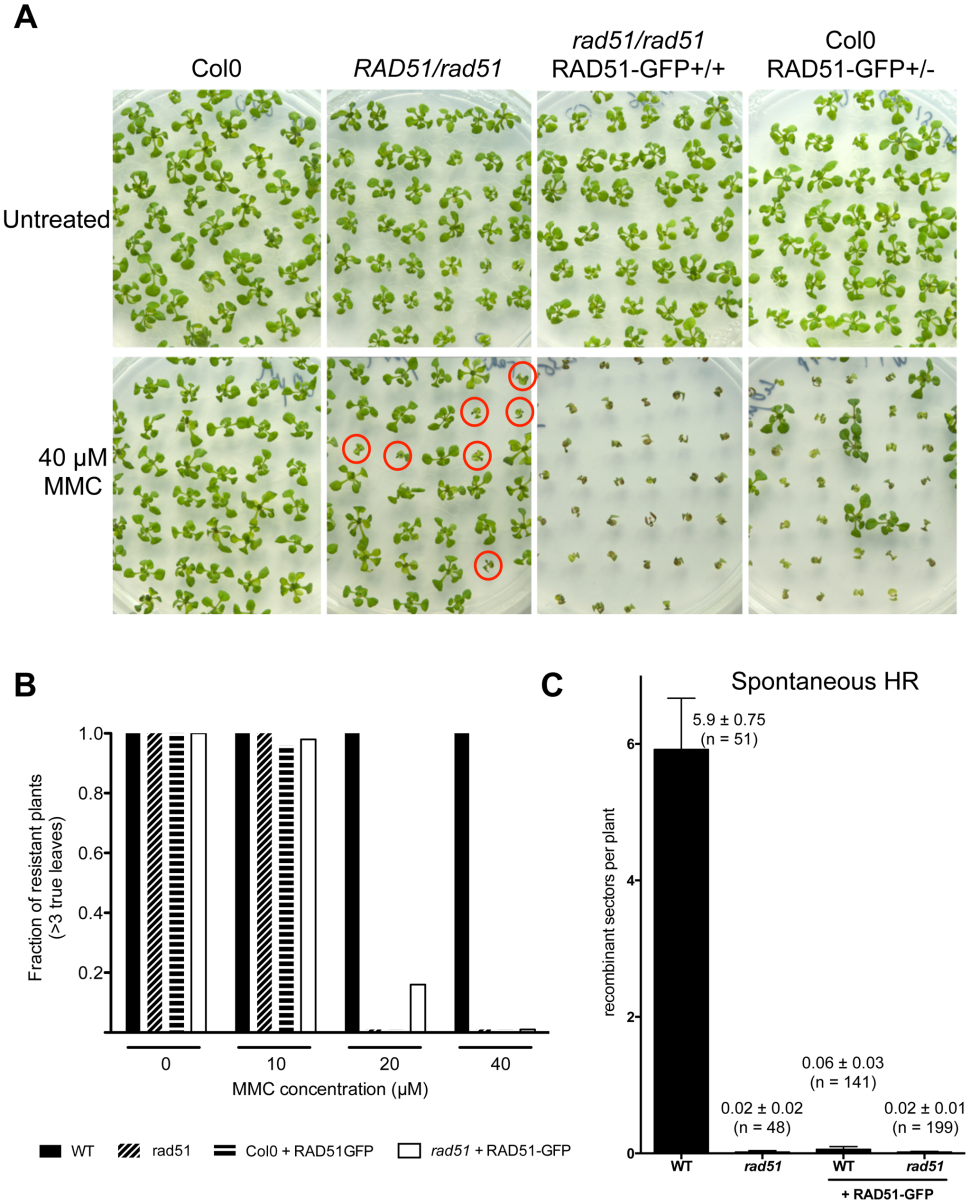
Defective mitotic recombination in plants expressing RAD51-GFP. (**A**) Sensitivity of plants expressing RAD51-GFP to DNA-damaging agent Mitomycin C (MMC). Two-week-old seedlings grown without or with 40 µM MMC are shown. *rad51* mutants are sterile and hence seeds of *RAD51/rad51* heterozygotes were used and only 1/4 of their progeny are homozygous and sensitive to MMC (depicted with red circles). Genotypes of the plants were confirmed by PCR and GFP visualisation. (**B**) Sensitivity of the seedlings (n = 50) was scored after 2 weeks (see Materials and Methods) and the fractions of resistant plants (plants with more than 3 true leaves) are shown. Black fill - Wild-type; diagonal fill - homozygous *rad51* mutants; horizontal fill - wild-type expressing RAD51-GFP protein; no fill - homozygous *rad51* mutants expressing the RAD51-GFP transgene. (**C**) Somatic homologous recombination is impaired in plants expressing RAD51-GFP fusion protein. Mean numbers of spontaneous GUS+ recombinant spots in wild-type plants and *rad51* mutants (PCR genotyped) expressing or not the RAD51-GFP fusion protein. Standard errors (SEM) and numbers of plants analysed (n) are indicated for each genotype. The wild-type plants with and without RAD51-GFP are sister plants from a population segregating the RAD51-GFP allele.

To confirm the function of the fusion protein in mitotic cells, we tested its capacity to complement the sensitivity of *rad51* mutant plants to the DNA cross-linking agent Mitomycin C (MMC). Wild-type and *rad51* mutant plants, carrying or not the RAD51-GFP fusion protein, were grown on solid media containing increasing concentrations of Mitomycin C and growth was scored after 2 weeks (Figure 4). As expected under these conditions, *rad51* mutants are highly sensitive while wild-type plants show little sensitivity to MMC (Figure 4A and B). Unexpectedly given the complementation in meiosis and the expression patterns in somatic cells, the RAD51-GFP protein does not complement the MMC hypersensitivity of *rad51* plants. It acts as a dominant negative with both wild-type and *rad51* mutant plants expressing the RAD51-GFP protein clearly hypersensitive to MMC (Figure 4A and B). This dominant negative phenotype implies that the fusion protein interferes with the proper functioning of the native RAD51 protein.

The importance of homologous recombination (HR) in the repair of DNA cross-links has led to the use of MMC hypersensitivity as an indirect test for recombination capacity in a number of organisms. Given the dominant negative MMC hypersensitivity conferred by RAD51-GFP, we also directly tested somatic homologous recombination in these plants using the previously described IU.GUS *in planta* recombination tester locus, consisting of an interrupted ß-glucuronidase (GUS) gene and a template GUS sequence for repair [35]–[37]. The IU.GUS recombination reporter locus was crossed into *rad51* mutant plants expressing the RAD51-GFP fusion protein and somatic HR frequencies (HRF) monitored in F3 progeny homozygous for IU.GUS. Figure 4C shows quantification of spontaneous somatic recombination in WT and *rad51* mutants, expressing or not the RAD51-GFP protein. As expected HR is severely reduced in *rad51*/*rad51* mutants (1 recombinant spot found in 48 plants) compared to wild-type plants, which have a mean of 5.9 recombination events per plant (SEM = 0.75; n = 51). The presence of RAD51-GFP in both WT and *rad51/rad51* plants resulted in levels of homologous recombination similar to those observed in the *rad51* mutant (Figure 4C). The RAD51-GFP protein is thus not functional in mitotic recombination and these results very clearly confirm the dominant-negative effect, with the presence of the RAD51-GFP protein reducing recombination of *RAD51/RAD51* plants to the level of *rad51/rad51* plants (100-fold reduction; Figure 4C).

Despite its ability to restore the fertility of *rad51* mutant plants, the RAD51-GFP protein is clearly defective for somatic homologous recombination. Presence of the RAD51-GFP protein creates a separation-of-function phenotype.

### RAD51-GFP assembles at sites of DNA breaks in mitosis and meiosis

**Figure 5:**
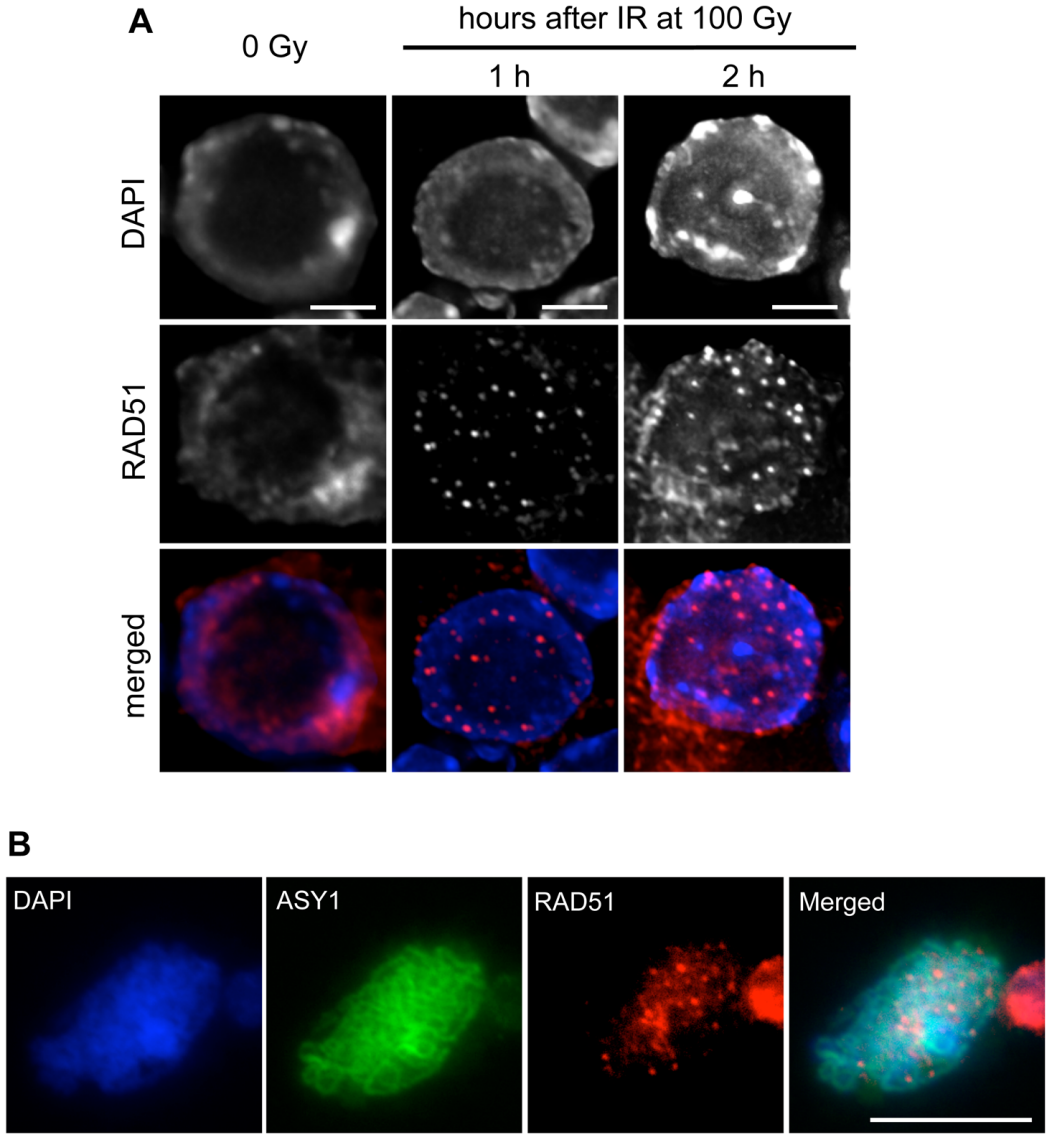
RAD51 foci are formed in root-tip nuclei and PMCs of *rad51* RAD51-GFP mutants. (**A**) Immunolocalisation of RAD51-GFP in nuclei of *rad51* RAD51-GFP plants fixed just before, and 1 or 2 hours after 100 Gy gamma-irradiation. Irradiation-induced RAD51-GFP foci are clearly visible in nuclei of the irradiated plants. DNA is stained with DAPI (blue) and RAD51-GFP foci (detected using an antibody against RAD51) are coloured in red. Images are collapsed Z-stack projections of a deconvoluted 3D image stack. (Scale Bar: 2 µm). (**B**) Immunolocalisation of RAD51-GFP in pollen mother cells (PMCs) of *rad51* RAD51-GFP mutants using an antibody against RAD51. DNA is stained with DAPI (blue) and RAD51-GFP foci are red. Synaptonemal complex protein ASY1 (in green) was used as a meiotic stage marker. Images are collapsed Z-stack projections of a deconvoluted 3D image stack. (Scale Bar: 10 µm).

The phenotypes conferred by the Arabidopsis RAD51-GFP fusion appear very similar to those recently described in yeast for the mutant *rad51-II3A* protein, which retains its ability to form nucleofilaments but has no joint molecule activity [22], [23]. Using immunocytology to detect RAD51 nucleofilaments as brightly staining nuclear foci, we tested whether the RAD51-GFP protein retains its ability to assemble at sites of DNA breaks induced by gamma-irradiation in *rad51*/*rad51* RAD51-GFP plants. We have recently shown that gamma-ray induced RAD51 foci are easily visualised in Arabidopsis using a dose of 100 Gy [38]. As expected, no foci and only diffuse anti-RAD51 nuclear staining were observed in root tip nuclei of unirradiated control plantlets (Figure 5A). Numerous foci were detected in nuclei from *rad51*/*rad51* RAD51-GFP root tips fixed one or two hours after 100 Gy of gamma-rays (Figure 5A), confirming that the ability of RAD51 to assemble at sites of DNA damage is retained in somatic cells. The radio-inducibility of the RAD51-GFP was confirmed by western blotting analyses after irradiation (Figure S1 and Protocol S1).

The ability of the RAD51-GFP fusion to assemble at DNA breaks in meiotic cells was confirmed by immunolocalisation of RAD51 and ASY1 in pollen mother cell nuclei of *rad51*/*rad51* RAD51-GFP plants, which show the expected numerous meiotic RAD51 foci (Figure 5B). Immunostaining of DMC1 protein in these nuclei revealed the expected presence of abundant DMC1 foci in the *rad51/rad51* RAD51-GFP plants (Figure S2).

### DMC1 alone is able to repair meiotic DSBs

The RAD51-GFP protein is thus present and forms nucleofilaments in *rad51*/*rad51* RAD51-GFP plants. Meiosis is normal and the severe, prophase I chromosome fragmentation of *rad51* mutants is fully complemented by the fusion protein. RAD51-GFP is properly expressed and forms the expected radio-induced foci in mitotic cells, but is catalytically non-functional in mitotic HR. The most straightforward explanation for these results is that DMC1 protein carries out meiotic DSB repair in *rad51*/*rad51* RAD51-GFP plants, and that it requires the presence of the (catalytically non-functional) RAD51-GFP protein. Should this be so, in the absence of DMC1, the RAD51-GFP protein should no longer be able to complement the *rad51* meiotic phenotype (chromosomal fragmentation).

**Figure 6:**
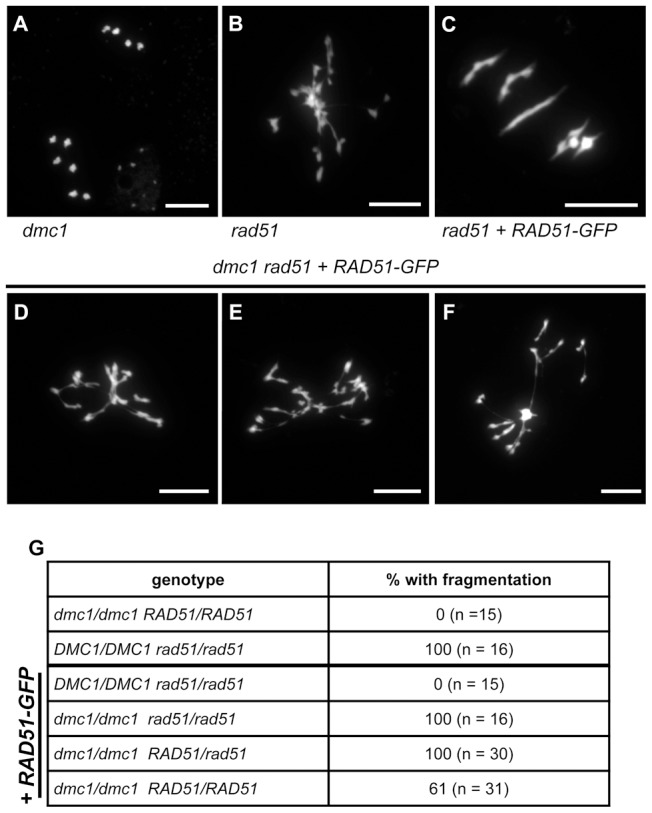
Absence of DMC1 in *rad51* RAD51-GFP mutants leads to extensive chromosome fragmentation. Meiotic spreads of PMCs showing ten intact univalents in *dmc1* mutants (**A**), strong chromosome fragmentation in *rad51* mutants (**B**) and five normal bivalents in *rad51* RAD51-GFP mutants (**C**). In *rad51* RAD51-GFP *dmc1* mutants (**D to F**) all meiotic nuclei show strong chromosome fragmentation. (Scale Bar: 10 µm). The proportions of meiocytes with fragmented chromosomes for each genotype are shown (**G**). (**A**) is metaphase II and (**B** to **G**) are metaphase I/Anaphase I.

We thus crossed *rad51/rad51* RAD51-GFP and *dmc1* mutant plants and identified the *rad51/rad51*, *dmc1/dmc1*, and *rad51/rad51 dmc1/dmc1* mutants in the F2, with and without RAD51-GFP. Observation of meiosis in pollen mother cells of these plants showed the expected ten intact univalents in *dmc1* (Figure 6A), and fragmented chromosomes in *rad51* metaphase I (Figure 6B). Meiosis progresses normally in *rad51* mutants expressing RAD51-GFP, with 5 bivalents visible at Metaphase I (Figure 6C). Extensive chromosome fragmentation and fusion were observed in 100% of the meiocytes (n = 16) of *rad51/rad51 dmc1/dmc1* RAD51-GFP plants (Figure 6D to F), clearly confirming that the meiotic DSB repair observed in *rad51*/*rad51* RAD51-GFP mutants is DMC1-dependent. In accord with the dominant negative mitotic phenotype, chromosome fragmentation was also observed in all meiocytes of *dmc1/dmc1 RAD51/rad51* plants expressing the RAD51-GFP fusion protein (n = 30. Figure 6G). The dominance was however incomplete, with fragmentation observed in only 61% of *dmc1/dmc1* RAD51/RAD51 meiocytes expressing the RAD51-GFP fusion protein (n = 31).

These data confirm that the complementation of the meiotic chromosome fragmentation and sterility of Arabidopsis *rad51* mutant plants by the RAD51-GFP protein is fully dependent upon the presence of DMC1. DMC1 is thus able to repair all meiotic DSB in Arabidopsis and depends upon the presence, not the strand exchange activity, of RAD51 to do so.

### Meiotic crossing-over rate is not affected in *rad51* RAD51-GFP plants

The RAD51 and DMC1 recombinases play essential roles in the repair of SPO11-induced meiotic DSB. The strikingly different phenotypes of Arabidopsis *rad51* and *dmc1* mutants however provide a clear illustration of their differing roles: the intact univalents in *dmc1* meiosis, and chromosome fragmentation in *rad51* meiosis showing that although RAD51 is able to repair all DSBs in the absence of DMC1, DMC1 cannot do so in the absence of RAD51. The absence of chiasmata in *dmc1* mutants furthermore confirming that interhomologue crossing-over is a DMC1-dependent process.

As shown above, meiotic DSB repair is carried out by the activity of DMC1 alone in *rad51* RAD51-GFP plants. We thus checked whether this results in elevated levels of meiotic interhomologue crossing-over in these plants. WT plants and *rad51/rad51* RAD51-GFP plants of the Columbia (*Col*) ecotype were crossed to a wild-type plant of the *Landsberg erecta* (*Ler*) ecotype, to yield *RAD51/rad51* plants heterozygotes for the RAD51-GFP transgene, and wild-type *RAD51/RAD51 Col/Ler* hybrids in the F1 (the dominant negative effect of the transgene allows analysis in F1 plants, see above and Figure 6D to 6F). Meiotic recombination was evaluated by analysing the segregation of markers in F2 populations originating from at least two F1 hybrid parents of each genotype. We measured crossing-over rates in two genetic intervals defined by insertion/deletion (INDEL) DNA sequence markers on chromosomes 1 and 3 (Table S1).

As seen in Table 1, no effect was observed on meiotic crossing-over rates in the *rad51* separation-of-function mutant for either of the two genetic intervals. This was confirmed through counting chiasmata in metaphase I of wild-type and *rad51/rad51* RAD51-GFP male meiocytes, which show means of 9.6 (SD = 0.5; n = 23) and 9.5 (SD = 0.5; n = 16) chiasmata per meiosis respectively. These results concord with those reported for the *rad51-II3A* yeast mutant and strongly suggest, as in yeast, that DMC1 is the catalytically active strand-exchange protein in Arabidopsis meiosis, and that RAD51 plays a supporting role [23].

**Table 1:** Meiotic recombination frequency (MRF) in WT and *rad51* RAD51-GFP mutant calculated from analyses of INDEL markers in F2 hybrids.

		WT		*rad51* RAD51-GFP		ratio
Interval	Physical distance (Mb)	N° of plants	MRF (%)	N° of plants	MRF (%)	
Chr1	3.8	175	0.14	165	0.14	**1.17**
		183	0.09	156	0.13	
Chr3	5.6	187	0.20	185	0.17	**0.97**
		175	0.21	183	0.23	

## Discussion

In most eukaryotes, meiotic recombination requires the co-operation of two strand-exchange proteins, RAD51 and DMC1. RAD51 is present and active in mitosis and meiosis while DMC1 is specific to meiosis. DMC1 is not absolutely necessary since several organisms do not possess a DMC1 orthologue (e.g. *Drosophila*, *Caenorhabditis elegans*, *Neurospora crassa* and *Sordaria macrospora*) [9]. Why meiosis necessitates two DNA strand-exchange proteins and what unique functions are accomplished by DMC1 remains however elusive. A key to this question comes perhaps from the recent description of the yeast *rad51-II3A* separation-of-function mutant, showing that the joint molecule forming activity of RAD51 is not needed for meiotic recombination [23]. In yeast, the strand-exchange activity of DMC1 alone is thus sufficient for meiotic recombination and the requirement for RAD51 is for the protein itself (as a nucleofilament) and not for its catalytic strand-exchange activity. We present here an analysis of an Arabidopsis RAD51-GFP fusion protein that produces analogous phenotypes to the yeast mutant, confirming the yeast results and extending them to the higher plant *Arabidopsis thaliana*, with the implication that these conclusions are potentially applicable in general to eukaryotes with a DMC1 homologue.

### Fusion of GFP to the C-terminus of Arabidopsis RAD51 results in a separation-of-function mutant

As a tool to further study the roles of RAD51 in plants, we tagged the Arabidopsis RAD51 protein with the Green Fluorescent Protein (GFP). A number of published studies of different organisms have made use of such tagged RAD51 proteins to analyse the *in vivo* localisation of RAD51 to chromatin (see Table S2 for a referenced list). These reports show that FP-tagged RAD51 proteins form foci in both mitotic and meiotic cells, that the kinetics of focus formation is similar to that of native RAD51 and accurately depicts the behaviour of the endogenous RAD51 proteins. The fusion of the fluorescent protein at the N- or C-termini of RAD51 does thus not affect the ability of the protein to assemble at DNA DSB. N-terminal fusions are able to complement (sometimes partially) the radiation sensitivity or inviability of yeast, human, chicken, *Ustilago maydis* and *Magnaporthae oryzae rad51* mutant cells (see Table S2). In contrast fusion of fluorescent proteins to the Rad51 C-terminus does not rescue the *rad51* mutant mitotic phenotype (tested in *S. pombe*, human and chicken cells - see Table S2). Furthermore a dominant negative effect was observed on repair of gamma-ray induced DSB when RAD51-GFP was expressed in human cells [39], although not for ultraviolet light hypersensitivity in *S. pombe* [40].

We show here that the Arabidopsis C-terminal RAD51-GFP fusion is properly expressed in dividing mitotic cells, that the fusion protein localises to the nucleus and forms the expected gamma-ray induced nuclear foci. This C-terminal fusion protein is not however functional in mitotic recombination and does not complement the MMC hypersensitivity of Arabidopsis *rad51* mutants. Furthermore, RAD51-GFP expression confers a dominant negative, *rad51*-like, recombination and MMC sensitivity phenotype on wild-type plantlets. As mentioned above, a human RAD51-GFP fusion protein also acts as a dominant negative in DSB repair and this phenotype presumably reflects the formation of inactive, mixed RAD51/RAD51-GFP nucleofilaments in these cells [39]. Although we do not have direct biochemical evidence, we favour the hypothesis that the RAD51-GFP fusion protein lacks strand-exchange activity by analogy with the phenotypes of the yeast *rad51-II3A* protein [23]. In yeast Rad51, mutation of three amino acids (R188, K361, and K371) in the low affinity DNA binding site inhibits the strand-exchange activity of the RAD51 protein, while leaving intact its capacity to form nucleofilaments [23]. These amino acids are conserved in the Arabidopsis RAD51 and two of them (R306, K316) are located in the C-terminal part of the protein (Figure S3). It is therefore possible that the steric effect caused by addition of the GFP affects the conformation of the RAD51 protein and/or the access of these amino acids to other proteins or DNA.

Considerably less is known concerning the activity of RAD51-GFP fusion proteins in meiosis. C-terminal fusions of RAD51 to GFP or RFP permit visualisation of RAD51 in meiotic cells of *Sordaria macrospora* [41]–[43]. Meiosis is normal in a wild-type Sordaria strain expressing the RAD51-GFP, with however a slight defect in sporulation (90 to 95% of viable spores instead of 100% in WT). In the absence of a *rad51* mutant, meiotic complementation by the fusion protein has not been tested directly 41,43. Given that no DMC1 orthologue has been identified in Sordaria, implying that both meiotic and mitotic recombination are catalysed by RAD51, this would argue that the fusion protein is not dominant negative, at least in meiosis.

### DMC1 is the active meiotic recombinase in Arabidopsis and RAD51 plays a supporting role

Recombination and interhomologue crossing-over are responsible for the physical recognition and linking of homologous chromosome pairs required to ensure proper chromosomal disjunction at the anaphase of the first meiotic division. The invasion of a template DNA duplex for the repair of a DSB by recombination is catalysed by RAD51-like strand-transfer recombinases and as is the case for many eukaryotes [9]. Arabidopsis has two of these: RAD51 active in meiosis and mitosis, and DMC1 which is meiosis-specific [44], [45]. Many studies specifically implicate the meiosis-specific DMC1 protein in meiotic crossing-over recombination with the homologous chromosome [2], [9], [46], as illustrated by the different meiotic phenotypes of Arabidopsis *rad51* and *dmc1* mutants. Absence of RAD51 in Arabidopsis meiosis leads to defects in chromosome pairing and synapsis, and to extensive chromosome fragmentation at meiotic zygotene/pachytene [28]–[32], [47]. Arabidopsis *dmc1* mutant plants have a strikingly different meiotic phenotype, with synapsis defects and absence of chiasmata leading to the random segregation of intact univalent chromosomes [27], [29]–[32], [47].

Notwithstanding our results in somatic cells showing that RAD51-GFP is inactive in mitotic recombination and cross-link repair, its presence fully complements the meiotic defects of *rad51* mutant plants. This essential activity is thus presumably furnished by the DMC1 protein in meiosis in *rad51* mutants expressing RAD51-GFP. Removal of DMC1 confirms this hypothesis, with *rad51*-like meiotic chromosome fragmentation observed in *dmc1 rad51* mutant plants expressing RAD51-GFP. The meiotic complementation of *rad51* mutant plants by the fusion protein is thus fully dependent on the presence of the DMC1 protein. Arabidopsis DMC1 is able to repair all meiotic DSB and to do so requires the presence of the RAD51 protein, not its activity.

DMC1 is thus the active strand-invasion enzyme in meiotic crossing-over recombination in both Arabidopsis and yeast. In addition to the results presented here, the role of RAD51 in supporting DMC1 is seen in the reduced numbers of meiotic DMC1 foci in *rad51* knockouts and in the phenotype of the *rad51-II3A* mutant [17], [18], [23], [31], [32]. A reduction of the fidelity of meiotic chromosome synapsis in the hypomorph Arabidopsis *rad51-2* mutant suggests a role for RAD51 supporting DMC1 function [29], as does the impaired RAD51 and DMC1 focus formation observed in Arabidopsis *brca2* plants [48]. In the absence of DMC1 however, significant levels of homologous pairing are observed in yeast [20], [21], [49], [50]. Similarly, traces of the synaptonemal complex central element (ZYP1 staining) and (limited) homologous chromosome synapsis of centromere-proximal regions are observed in the Arabidopsis *dmc1* mutant [29], [30], [32].

The RAD51 nucleofilament thus plays a crucial role in regulating DMC1 activity but its strand-exchange activity must be inhibited in meiosis. In *S. cerevisiae*, restriction of the activity of RAD51 involves the action of the meiosis-specific protein Hed1. Hed1 down-regulates the activity of RAD51 to disfavour the use of the sister chromatid and hence favour DMC1-dependent inter-homologue recombination [51]–[53]. No Hed1 homologue has been identified in plants, but it seems likely that such a regulator exists. Recent work shows the importance of the ATR (Mec1) in regulating DMC1 filament formation in Arabidopsis, with absence of ATR in the Arabidopsis *rad51* mutant permitting DMC1 assembly and subsequent synapsis, meiotic DSB repair and crossing-over formation [31]. DMC1 inter-homologue recombination in Arabidopsis is also controlled by ASY1 and ASY3, the Arabidopsis homologues of Hop1 and Red1 [31], [54], [55]. Arabidopsis DMC1 is thus clearly able to catalyse meiotic recombination using both sister or non-sister chromatid templates. In the absence of RAD51 this is however relatively inefficient as, in contrast to the separation-of-function mutant described here, *atr rad51* double mutants still show significant chromosome fragmentation in meiosis [31].

Does this mean that DMC1 catalyses all strand-invasion in wild-type meioses? Either all meiotic recombination is catalysed by DMC1 with support from the RAD51 nucleofilament, or both DMC1 and RAD51 act catalytically in strand-invasion. It is not possible to distinguish between these two possibilities at this time, however the absence of an effect on crossing-over in both yeast and Arabidopsis is intriguing, given the significant variation in relative numbers of meiotic DSB and crossing-overs, with a ratio of 1.8 in yeast and 25–30 fold more DSB than crossing-overs in Arabidopsis (a high ratio is also seen in mice, with 15-fold more DSB than crossing-overs - see review by [24]). This would favour the idea of a supporting role for RAD51 in promoting DMC1 activity, and this was confirmed directly by *in vitro* experiments showing that both yeast RAD51 and rad51-II3A proteins stimulate the D-loop forming activity of DMC1 in the presence of the Mei5-Sae3 [23]. That RAD51 strand-transfer activity does play at least a minor “fail-safe” role in yeast meiosis is however suggested by the observation of a delay in the appearance of joint molecules and a slight reduction in sporulation efficiency (from 99 to 87%) in *rad51-II3A* [23]. No orthologues of Mei5 or Sae3 have been identified as yet in Arabidopsis [31], but it seems probable that they, or proteins of equivalent function exist.

Working with *Arabidopsis thaliana*, we describe here a RAD51-GFP fusion protein that lacks DNA repair activity but retains the capacity to assemble at DNA breaks. This protein fully complements the meiotic chromosomal fragmentation and sterility of Arabidopsis *rad51* mutants, and we show that this depends upon DMC1. Even though DMC1 is the only active recombinase in the absence of RAD51 catalytic activity, no effect on genetic map distance was observed in complemented *rad51* plants. The presence of inactive RAD51 nucleofilaments is thus able to fully support meiotic DSB repair and normal levels of crossing-over by DMC1 in Arabidopsis.

## Materials And Methods

### Plant material and growth conditions

The *Arabidopsis thaliana rad51* (AT5G20850) and *dmc1* (AT3G22880) mutants used in this work have been previously described [27], [28].

Plants were grown under standard conditions: seeds were stratified in water at 4°C for 2 days and grown on soil or *in vitro* on 0.8% agar plates, 1% sucrose and 0.5× Murashige and Skoog salts (M0255; Duchefa Biochemie). Plants were then cultivated in a greenhouse or growth chamber with a 16/8 hour light/dark cycle, temperature 23°C and 45% to 60% relative humidity.

### Cloning of RAD51 and plant transformation

For translational GFP fusions, the genomic region without stop codon and a 1036 bp 5′ upstream sequence of RAD51 was amplified (forward primer TGATTAGCATTTAGCGTCAAG and reverse primer ATCCTTGCAATCTGTTACACC), inserted into pDONR221 and verified by sequencing. The complete fragment was then cloned into the GATEWAY destination vector pB7FWG2 in which the 35S promoter was removed with a SacI/SpeI digest [56]. The plasmid was inserted in an *Agrobacterium tumefaciens* C58C1 strain and used to transform wild-type and *rad51* mutant plants by the floral dip method [57].

### Mitomycin C sensitivity and somatic homologous recombination assay

For the Mitomycin C sensitivity assay, seeds were surface-sterilised and sown onto solid medium containing 0.5× Murashige and Skoog salts, 1% sucrose, 0.8% agar and 0, 10, 20 or 40 µM Mitomycin C (SIGMA). After stratification for 2 days at 4°C, plants were grown for two weeks and sensitivity analysed as previously described [58], [59]. Plants with more than three true leaves were considered as resistant [58], [59].

The IU.GUS *in planta* recombination tester locus consisting of an interrupted ß-glucuronidase (GUS) gene and an internal repair template GUS sequence [35]–[37] was used to determine the rate of spontaneous somatic homologous recombination. Seeds were surface-sterilised, stratified at 4°C for 2 days and grown in petri dishes on 0.8% w/v agar, 1% w/v sucrose and 0.5× Murashige and Skoog salts for 2 weeks. Seedlings were then harvested and incubated in staining buffer containing 50 mM sodium phosphate buffer (pH 7.2), 0.2% v/v Triton X100, and 2 mM X-Gluc dissolved in N,N-dimethylformamide. Plants were then infiltrated under vacuum for 15 min and incubated 24 hours at 37°C. Staining solution was replaced with 70% ethanol to remove chlorophyll and blue spots counted under a dissecting microscope.

### Analysis of meiotic recombination rate

Wild-type and *rad51* RAD51-GFP mutant plants (*Col* ecotype) were crossed with wild-type *Landsberg erecta* ecotype *(Ler)* plants. RAD51/rad51 heterozygotes carrying the RAD51-GFP (also heterozygous), and RAD51/RAD51 wild-type F1, *Col/Ler* hybrids were selected. Meiotic recombination rates were monitored in the F2 segregating populations by INDEL marker genotyping. For genotyping, seeds from the F2 populations were surface-sterilised and grown in vitro on 0.5× MS, 1% sucrose, 0.8% agar for two-weeks. Individual seedlings were harvested, DNA extracted using NaCl method and samples genotyped by PCR followed by analysis on 2% agarose gels.

### Chromosome spreads

Meiotic chromosome spreads were prepared according to Ross [60] with the modifications introduced by Fransz [61]. Whole inflorescences were fixed in ice-cold ethanol/glacial acetic acid (3∶1) for 3×30 min and stored at −20°C until further use. Immature flower buds were rinsed twice at room temperature in distilled water for 5 min followed by two washes in 1× citrate buffer for 5 min. Buds of appropriate size were selected under a binocular microscope and incubated for 3.5 h on a slide in 100 µl of enzyme mixture (0.3% w/v cellulase (Sigma), 0.3% w/v pectolyase (Sigma) and 0.3% cytohelicase (Sigma) in a moist chamber at 37°C. Each bud was then softened for 1 minute in 15 µl 60% acetic acid on a microscope slide at 45°C, fixed with ice-cold ethanol/glacial acetic acid (3∶1) and air dried. Finally, slides were mounted in Vectashield mounting medium with DAPI (1.5 µg.ml^−1^; Vector Laboratories Inc., http://www.vectorlabs.com/).

### RAD51 immunostaining in root tip nuclei

Five day-old seedlings were irradiated with a dose of 100 Gy from a ^137^Cs source according to Charbonnel et al. (2010). Preparation and immunostaining of nuclei were performed as previously described [62], except that slides were incubated with primary antibody (1∶100) for 24 hours at 4°C. The RAD51 antibody used in this study has been previously described and was raised in rabbit [63].

### Immunolocalisation in pollen mother cells

Immunolocalisation of proteins in pollen mother cells was performed as described previously [33], with the modifications introduced by Kurzbauer et al [31]. The anti-ASY1 raised in Guinea-Pig [33], [64] was used at a dilution of 1∶250. The anti-ZYP1 raised in rat [34] was used at a dilution of 1∶250. The anti-RAD51 raised in rabbit [63] was used at a dilution of 1∶100, and the anti-DMC1 raised in rabbit [65] was used at a dilution of 1∶20.

### Microscopy

All observations were made with a motorised Zeiss AxioImager.Z1 epifluorescence microscope (Carl Zeiss AG, Germany) using a PL Apochromat 100X/1.40 oil objective. Photographs were taken with an AxioCam Mrm camera (Carl Zeiss AG, Germany) and appropriate Zeiss filter sets adapted for the fluorochromes used: filter set 25HE (DAPI), filter set 38HE (Alexa 488), and filter set 43HE (Alexa 596). Image stacks were captured in three dimensions (x, y, z) and further deconvoluted with the deconvolution module (theoretical PSF, iterative algorithm) of AxioVision 4.6.2 software (Carl Zeiss AG, Germany). For presentation, the pictures are collapsed Z-stack projections obtained using the Extended-focus module (projection method) of the AxioVision 4.6.2 software.

## Supporting Information

Figure S1 RAD51-GFP protein is induced by gamma-irradiation. Total proteins were extracted from seedlings collected at the indicated time after treatment with 100 Gy of gamma-rays and RAD51-GFP abundance measured. (**A**) Western blot (40 µg protein/lane) showing the time-dependent changes in RAD51-GFP abundance in 5 day-old seedlings irradiated with 100 Gy of gamma-rays. (**B**) Quantification of RAD51-GFP protein. Numbers above bars indicate fold-induction relative to untreated samples (NT).
(PDF)

Figure S2 Immunolocalisation of DMC1 in *rad51* RAD51-GFP mutants. Male meiocytes stained with DAPI (blue), anti-AtASY1 antibody (green), and anti-DMC1 antibody (red). ASY1 extends along the entire length of the chromosome axes and numerous DMC1 foci are visible on the chromosomes (Scale bar = 10 µm.).
(PDF)

Figure S3 Sequence alignment of RAD51 proteins. Alignment of RAD51 from *Saccharomyces cerevisiae* (ScRAD51) and *Arabidopsis thaliana* (AtRAD51) was generated using ClustalW. Numbers indicate amino acid positions. Red boxes indicate the three critical amino acids in yeast rad51-II3A mutant and their conservation in Arabidopsis. Under the sequences, asterisks, colons and full stops indicate identical, conserved and semi-conserved residues respectively.
(PDF)

Table S1 List of PCR markers between Col and Ler. For each marker, the corresponding BAC clone, primers, size of the Columbia and Landsberg products, and the physical distance of the intervals are listed.
(PDF)

Table S2 Studies that have used fluorescent protein to investigate RAD51 foci formation and function. FP: Fluorescent protein. Ns: Not shown; CHO: Chinese Hamster Ovary; ES cells: Embryonic Stem cells. ^1^ *YFP was fused to a rad51-I345T* mutant encoding protein with a higher affinity for DNA than wild-type RAD51.
(PDF)

Protocol S1 Western blot analyses.
(PDF)
