# Supplementary figures and images for: Meiotic Recombination in Arabidopsis Is Catalysed by DMC1, with RAD51 Playing a Supporting Role
Source: PLoS Genet. 2013 Sep 26;9(9):e1003787. doi: 10.1371/journal.pgen.1003787 (PMC3784562; doi:10.1371/journal.pgen.1003787)

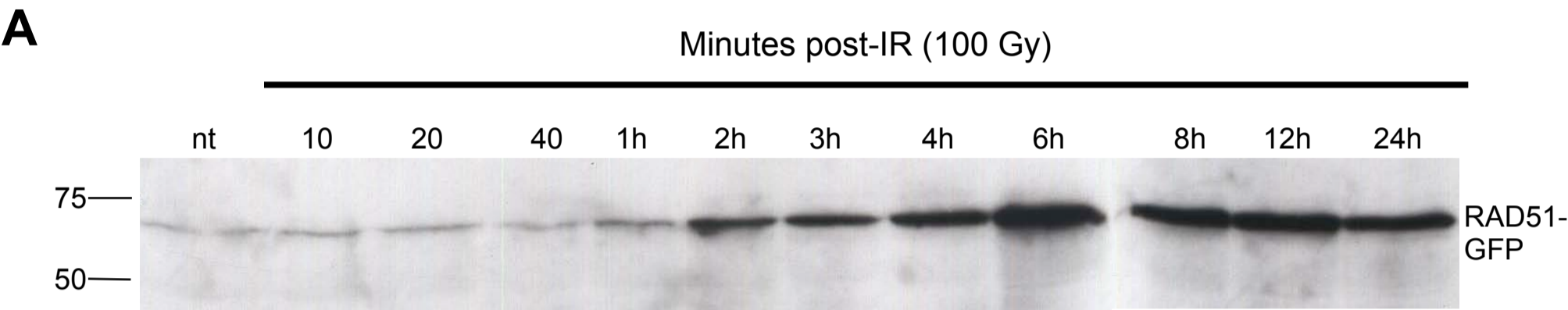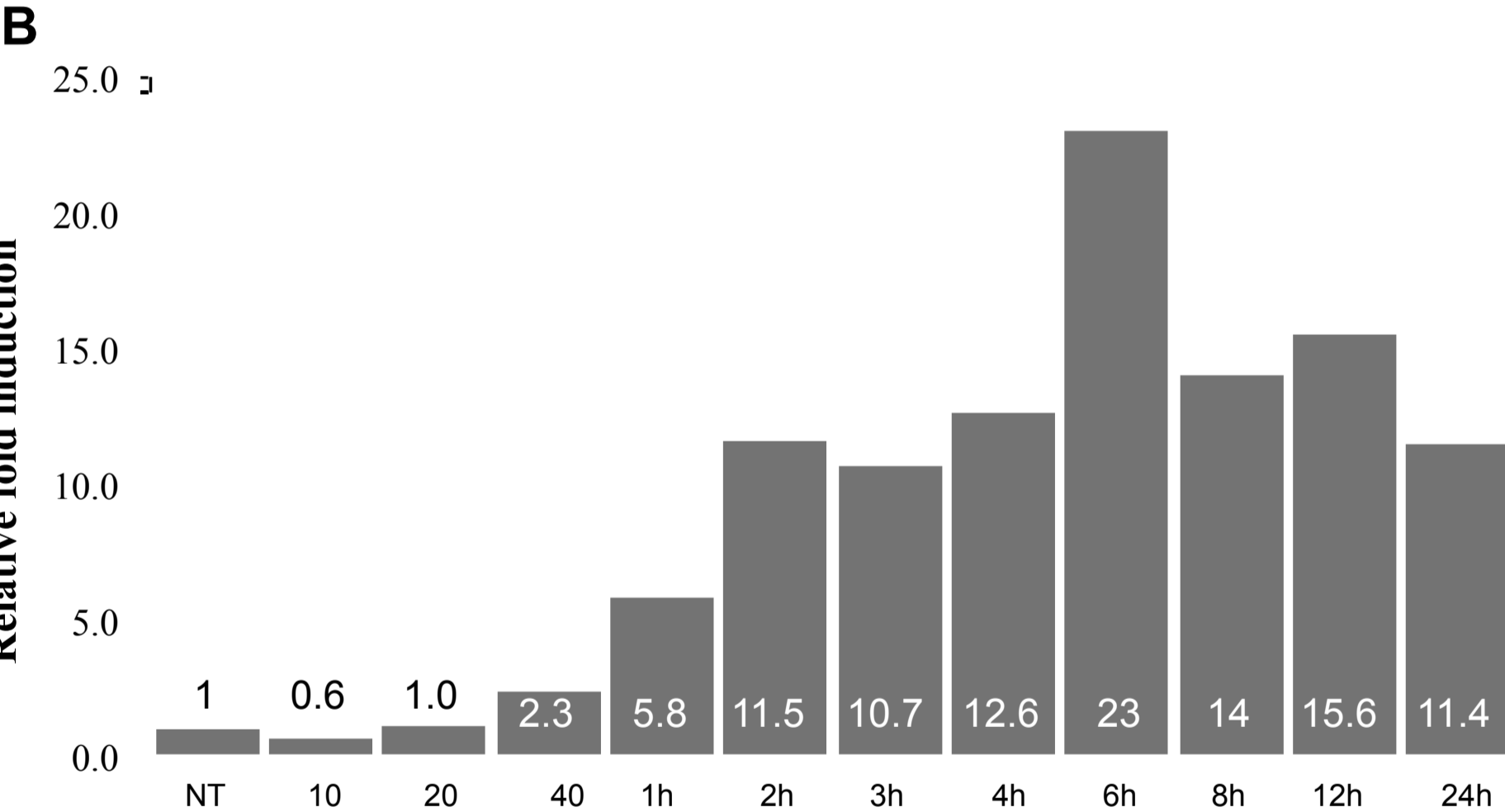

Supplement: Figure S1 — RAD51-GFP protein is induced by gamma-irradiation. Total proteins were extracted from seedlings collected at the indicated time after treatment with 100 Gy of gamma-rays and RAD51-GFP abundance measured. (A) Western blot (40 µg protein/lane) showing the time-dependent changes in RAD51-GFP abundance in 5 day-old seedlings irradiated with 100 Gy of gamma-rays. (B) Quantification of RAD51-GFP protein. Numbers above bars indicate fold-induction relative to untreated samples (NT). (PDF) [file pgen.1003787.s001.pdf]

DAPI/ASY1/DMC1

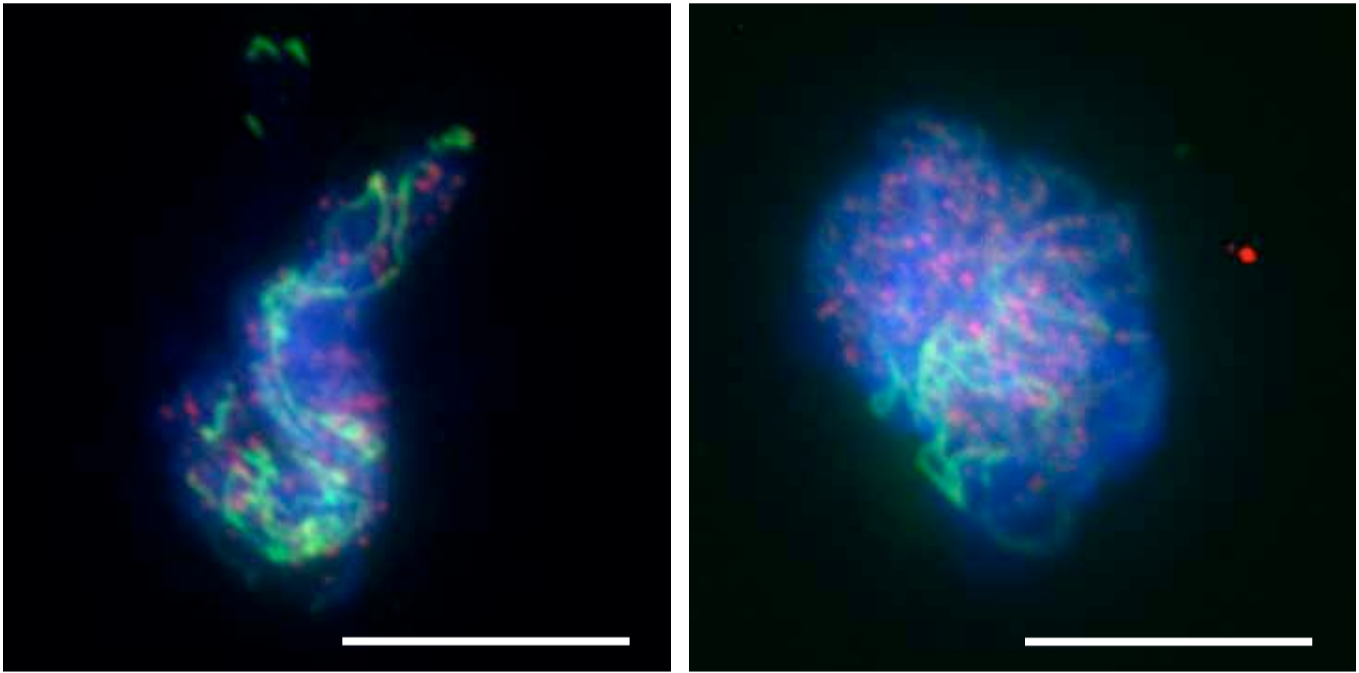

Supplement: Figure S2 — Immunolocalisation of DMC1 in rad51 RAD51-GFP mutants. Male meiocytes stained with DAPI (blue), anti-AtASY1 antibody (green), and anti-DMC1 antibody (red). ASY1 extends along the entire length of the chromosome axes and numerous DMC1 foci are visible on the chromosomes (Scale bar = 10 µm.). (PDF) [file pgen.1003787.s002.pdf]
