# Supplementary material for: Meiotic Recombination in Arabidopsis Is Catalysed by DMC1, with RAD51 Playing a Supporting Role
Source: PLoS Genet. 2013 Sep 26;9(9):e1003787. doi: 10.1371/journal.pgen.1003787 (PMC3784562; doi:10.1371/journal.pgen.1003787)
Supplement: Figure S3 — Sequence alignment of RAD51 proteins. Alignment of RAD51 from Saccharomyces cerevisiae (ScRAD51) and Arabidopsis thaliana (AtRAD51) was generated using ClustalW. Numbers indicate amino acid positions. Red boxes indicate the three critical amino acids in yeast rad51-II3A mutant and their conservation in Arabidopsis. Under the sequences, asterisks, colons and full stops indicate identical, conserved and semi-conserved residues respectively. (PDF) [file pgen.1003787.s003.pdf]

Da Ines_FigS3

```
ScRAD51         MSQVQEQHISESQLQYGNGSLMSTVPADLSQSVVDGNGNGSSEDIEATNGSGDGGGLQEQ 60
AtRAD51         ---------------------MTTMEQRRNQNAVQ-----QQDDEETQHGP--------- 25
                                     *:*:    .*..*:     ..:* *: :*.

ScRAD51         AEAQGEMEDEAYDEAALGSFVPIEKLQVNGITMADVKKLRESGLHTAEAVAYAPRKDLLE 120
AtRAD51         --------------------FPVEQLQAAGIASVDVKKLRDAGLCTVEGVAYTPRKDLLQ 65
                                    .*:*:**. **: .******::** *.*.***:******:

ScRAD51         IKGISEAKADKLLNEAARLVPMGFVTAADFHMRRSELICLTTGSKNLDTLLGGGVETGSI 180
AtRAD51         IKGISDAKVDKIVEAASKLVPLGFTSASQLHAQRQEIIQITSGSRELDKVLEGGIETGSI 125
                *****:**.**::: *::***:**.:*:::* :*.*:* :*:**::**.:* **:*****

ScRAD51         TELFGEFRTGKSQLCHTLAVTCQIPLDIGGGEGKCLYIDTEGTFRPVRLVSIAQRFGLDP 240
AtRAD51         TELYGEFRSGKTQLCHTLCVTCQLPMDQGGGEGKAMYIDAEGTFRPQRLLQIADRFGLNG 185
                ***:****:**:******.****:*:* ******.:***:****** **:.**:****:

ScRAD51         DDALNNVAYARAYNADHQLRLLDAAAQMMSESRFSLIVVDSVMALYRTDFSGRGELSARQ 300
AtRAD51         ADVLENVAYARAYNTDHQSRLLLEAASMMIETRFALLIVDSATALYRTDFSGRGELSARQ 245
                 *.*:*********:*** ***  **.** *:**:*::***. *****************

ScRAD51         MHLAKFMRALQRLADQFGVAVVVTNQVVAQVDGGMAFNPDPKKPIGGNIMAHSSTTRLGF 360
AtRAD51         MHLAKFLRSLQKLADEFGVAVVITNQVVAQVDGSALFAGPQFKPIGGNIMAHATTTRLAL 305
                ******:*:**:***:******:**********.  *     **********::****.:

ScRAD51         KKGKGCQRLCKVVDSPCLPEAECVFAIYEDGVGDPREEDE 400
AtRAD51         RKGRAEERICKVISSPCLPEAEARFQISTEGVTDCKD--- 342
                :**:. :*:***:.********. * *  :** * ::
```
