# Supplementary material for: Meiotic Recombination in Arabidopsis Is Catalysed by DMC1, with RAD51 Playing a Supporting Role
Source: PLoS Genet. 2013 Sep 26;9(9):e1003787. doi: 10.1371/journal.pgen.1003787 (PMC3784562; doi:10.1371/journal.pgen.1003787)
Supplement: Table S1 — List of PCR markers between Col and Ler. For each marker, the corresponding BAC clone, primers, size of the Columbia and Landsberg products, and the physical distance of the intervals are listed. (PDF) [file pgen.1003787.s004.pdf]

Supplemental Table S1. List of PCR markers between Col and Ler.

For each marker, the corresponding Bacterial Artificial Chromosome, primers, size of the Columbia and Landsberg products, and the physical distance of the intervals are listed.

Marker Name	Chr	BAC	Forward primer sequence	Reverse primer sequence	Marker type	Col / Ler length	Physical distance (Mb)
C1M1	1	F10O3	TGCATTGCATTTTGGATTA	CCGAAAAATATCAAGTGTTG	InDel	283 / 234	3.8
C1M2	1	F3F19	CGACGCATCAACAATGTACT	GCTTGGAATCGGCTTCTCTT	InDel	326 / 249	
C3M1	3	F4P13	AATCTAACAAGGCAAAAGGTC	CTCGCAACCAACCTACAAG	InDel	215 / 164	5.6
C3M2	3	T2O4	GAACTGCAATTGATGGAAACC	TGGAAATGATACTCTACTCGG	InDel	256 / 157	
