# Supplementary material for: Meiotic Recombination in Arabidopsis Is Catalysed by DMC1, with RAD51 Playing a Supporting Role
Source: PLoS Genet. 2013 Sep 26;9(9):e1003787. doi: 10.1371/journal.pgen.1003787 (PMC3784562; doi:10.1371/journal.pgen.1003787)
Supplement: Table S2 — Studies that have used fluorescent protein to investigate RAD51 foci formation and function. FP: Fluorescent protein. Ns: Not shown; CHO: Chinese Hamster Ovary; ES cells: Embryonic Stem cells. 1 YFP was fused to a rad51-I345T mutant encoding protein with a higher affinity for DNA than wild-type RAD51. (PDF) [file pgen.1003787.s005.pdf]

Supplemental Table S2. Studies that have used fluorescent protein to investigate RAD51 foci formation and function. FP: Fluorescent protein. Ns: Not shown; CHO: Chinese Hamster Ovary; ES cells: Embryonic Stem cells.

Reference	FP	N- or C-Term. fusion	Complementation	Foci (somatic cells)	Foci (Meiotic cells)	Organism
[1-3]	YFP	N-terminal	Partial complementation	+	Ns	*Saccharomyces cerevisiae*
[4, 5]	YFP	N-terminal	Complementation[1]	+	Ns	*Saccharomyces cerevisiae*
[6, 7]	GFP	N-terminal	Mutant not shown but no dominant negative effect	+	Ns	CHO cells and mouse ES cells
[8]	GFP, YFP, CFP	N-terminal	Mutant not shown but no dominant negative effect	+	Ns	CHO cells and mouse ES cells
[9]	GFP	N-terminal	Complementation and no dominant negative effect	+	Ns	mouse ES cells
[10]	GFP	N-terminal	Complementation	+	Ns	Human cells (HEK293)
[11]	GFP	N-terminal	Complementation	+	Ns	Human cells (HEK293) and Chicken DT40 cells
[12]	GFP	N-terminal	Complementation	+	Ns	Human cells (HEK293)
[13-15]	GFP	N-terminal	Partial complementation and no dominant negative effect	+	Ns	*Ustilago maydis*
[16]	GFP	N-terminal	Complementation	+	Ns	*Magnaporthe oryzae*
[17]	CFP	C-terminal	Ns	+	Ns	*Saccharomyces cerevisiae*
[18]	CFP	C-terminal	Ns	+	Ns	*Saccharomyces cerevisiae*
[19, 20]	CFP	C-terminal	Does not complement	+	Ns	*Schizosaccharomyces pombe*
[11]	GFP	C-terminal	C-terminal fusion does not complement *rad51-/-*	Ns	Ns	Chicken DT40 cells
[12]	GFP	C-terminal	C-terminal fusion does not complement and has dominant negative effect	Ns	Ns	Human cells (HEK293)
[21]	GFP	C-terminal	Ns	+	Ns	*Trypanosoma brucei*
[22-24]	GFP, RFP	C-terminal	Ns	ns	+	*Sordaria macrospora*

[1]*YFP was fused to a rad51-I345T* mutant encoding protein with a higher affinity for DNA than wild-type RAD51

REFERENCES

1. Lisby M, Barlow JH, Burgess RC, Rothstein R (2004) Choreography of the DNA damage response: spatiotemporal relationships among checkpoint and repair proteins. Cell 118: 699-713.
2. Kaiser GS, Germann SM, Westergaard T, Lisby M (2011) Phenylbutyrate inhibits homologous recombination induced by camptothecin and methyl methanesulfonate. Mutation research 713: 64-75.

3. Torres-Rosell J, Sunjevaric I, De Piccoli G, Sacher M, Eckert-Boulet N, et al. (2007) The Smc5-Smc6 complex and SUMO modification of Rad52 regulates recombinational repair at the ribosomal gene locus. Nature cell biology 9: 923-931.
4. Fung CW, Mozlin AM, Symington LS (2009) Suppression of the double-strand-break-repair defect of the Saccharomyces cerevisiae rad57 mutant. Genetics 181: 1195-1206.
5. Fasullo M, Chen Y, Bortcosh W, Sun M, Egner PA (2010) Aflatoxin B(1)-Associated DNA Adducts Stall S Phase and Stimulate Rad51 foci in Saccharomyces cerevisiae. Journal of nucleic acids 2010: 456487.
6. Essers J, Houtsmuller AB, van Veelen L, Paulusma C, Nigg AL, et al. (2002) Nuclear dynamics of RAD52 group homologous recombination proteins in response to DNA damage. The EMBO journal 21: 2030-2037.
7. Banath JP, Klokov D, MacPhail SH, Banuelos CA, Olive PL (2010) Residual gammaH2AX foci as an indication of lethal DNA lesions. BMC cancer 10: 4.
8. van Veelen LR, Essers J, van de Rakt MW, Odijk H, Pastink A, et al. (2005) Ionizing radiation-induced foci formation of mammalian Rad51 and Rad54 depends on the Rad51 paralogs, but not on Rad52. Mutation research 574: 34-49.
9. Kim TM, Ko JH, Hu L, Kim SA, Bishop AJ, et al. (2012) RAD51 mutants cause replication defects and chromosomal instability. Molecular and cellular biology 32: 3663-3680.
10. Pellegrini L, Yu DS, Lo T, Anand S, Lee M, et al. (2002) Insights into DNA recombination from the structure of a RAD51-BRCA2 complex. Nature 420: 287-293.
11. Yu DS, Sonoda E, Takeda S, Huang CL, Pellegrini L, et al. (2003) Dynamic control of Rad51 recombinase by self-association and interaction with BRCA2. Molecular cell 12: 1029-1041.
12. Forget AL, Loftus MS, McGrew DA, Bennett BT, Knight KL (2007) The human Rad51 K133A mutant is functional for DNA double-strand break repair in human cells. Biochemistry 46: 3566-3575.
13. Kojic M, Mao N, Zhou Q, Lisby M, Holloman WK (2008) Compensatory role for Rad52 during recombinational repair in Ustilago maydis. Molecular microbiology 67: 1156-1168.
14. Kojic M, Zhou Q, Lisby M, Holloman WK (2006) Rec2 interplay with both Brh2 and Rad51 balances recombinational repair in Ustilago maydis. Molecular and cellular biology 26: 678-688.
15. Mielnichuk N, Sgarlata C, Perez-Martin J (2009) A role for the DNA-damage checkpoint kinase Chk1 in the virulence program of the fungus Ustilago maydis. Journal of cell science 122: 4130-4140.
16. Ndindeng SA, Miki S, Abe A, Asano K, Sone T (2010) EGFP-Rhm51 foci enable the visualization and enumeration of DNA double-strand breaks in Magnaporthe oryzae. J Gen Plant Pathol 76: 377-381.
17. Lettier G, Feng Q, de Mayolo AA, Erdeniz N, Reid RJ, et al. (2006) The role of DNA double-strand breaks in spontaneous homologous recombination in S. cerevisiae. PLoS genetics 2: e194.
18. Moore DM, Karlin J, Gonzalez-Barrera S, Mardiros A, Lisby M, et al. (2009) Rad10 exhibits lesion-dependent genetic requirements for recruitment to DNA double-strand breaks in Saccharomyces cerevisiae. Nucleic acids research 37: 6429-6438.
19. Akamatsu Y, Tsutsui Y, Morishita T, Siddique MS, Kurokawa Y, et al. (2007) Fission yeast Swi5/Sfr1 and Rhp55/Rhp57 differentially regulate Rhp51-dependent recombination outcomes. The EMBO journal 26: 1352-1362.
20. Okamoto SY, Sato M, Toda T, Yamamoto M (2012) SCF ensures meiotic chromosome segregation through a resolution of meiotic recombination intermediates. PloS one 7: e30622.
21. Glover L, Horn D (2012) Trypanosomal histone gammaH2A and the DNA damage response. Molecular and biochemical parasitology 183: 78-83.
22. Tesse S, Storlazzi A, Kleckner N, Gargano S, Zickler D (2003) Localization and roles of Ski8p protein in Sordaria meiosis and delineation of three mechanistically distinct steps of meiotic homolog juxtaposition. Proceedings of the National Academy of Sciences of the United States of America 100: 12865-12870.
23. Storlazzi A, Gargano S, Ruprich-Robert G, Falque M, David M, et al. (2010) Recombination proteins mediate meiotic spatial chromosome organization and pairing. Cell 141: 94-106.
24. Storlazzi A, Tesse S, Ruprich-Robert G, Gargano S, Poggeler S, et al. (2008) Coupling meiotic chromosome axis integrity to recombination. Genes & development 22: 796-809.
