# Supplementary material for: Meiotic Recombination in Arabidopsis Is Catalysed by DMC1, with RAD51 Playing a Supporting Role
Source: PLoS Genet. 2013 Sep 26;9(9):e1003787. doi: 10.1371/journal.pgen.1003787 (PMC3784562; doi:10.1371/journal.pgen.1003787)
Supplement: Protocol S1 — Western blot analyses. (PDF) [file pgen.1003787.s006.pdf]

Supporting Protocol

Western blot analyses

Five day-old seedlings were irradiated with a dose of 100 Gy from a ^{137}Cs source according to [62]. At different time point after irradiation (10', 20', 40', 1h, 2h, 3h, 4h, 6h, 8h, 12h, and 24h), about 50 seedlings were frozen in liquid nitrogen. For total protein extraction, samples were ground in 100 µL extraction buffer (0.1 M Tris-HCl pH 7.5, 20% Glycerol, 2 mM EDTA, 1 mM DTT, 0.2% NP40 Nonidet, 1X complete Protease inhibitor cocktail (Roche)) and centrifuged at 4°C for 30 min at 20 000 g. Total protein content in the supernatant was carefully determined using the Bradford assay (BioRad). 40 µg of proteins were denatured in the presence of 1X Laemmli Buffer at 95°C for 5 min (Laemmli 1970). Proteins were electrophoresed on 10% SDS-polyacrylamide gels (Laemmli 1970) and transferred to nitrocellulose membrane. Equal protein loading was carefully controlled by reversible ponceau staining of the nitrocellulose membrane. RAD51-GFP proteins were detected with a mouse anti-GFP antibody at a dilution of 1:1000 (Roche, 11 814 460 001) followed by goat anti-mouse HRP-conjugated secondary antibody (1:10000 dilution; G&E Healthcare, Freiburg, Germany) and electrochemiluminescence detection ((ECL Plus; GE Healthcare). For quantitation, the intensity of each band was corrected for background and measured using Quantity One software (BioRad).

Reference

Laemmli UK. 1970. Cleavage of structural proteins during the assembly of the head of bacteriophage T4. *Nature* **227**: 680-685.
